# In vitro magnetosome remineralization for silver-magnetite hybrid magnetosome biosynthesis and used for healing of the infected wound

**DOI:** 10.1186/s12951-022-01532-4

**Published:** 2022-08-06

**Authors:** Junjie Xu, Shijiao Ma, Wei Zhang, Lina Jia, Haolan Zheng, Pang Bo, Xue Bai, Hongyan Sun, Lei Qi, Tongwei Zhang, Chuanfang Chen, Feng Li, Fumihito Arai, Jiesheng Tian, Lin Feng

**Affiliations:** 1 School of Mechanical Engineering and Automation, Beihang University, Beijing, 100083 China; 2 State Key Laboratory of Agrobiotechnology, College of Biological Sciences, China Agricultural University, Beijing, 100193 China; 3 State Key Laboratory of Ophthalmology, School of Biomedical Engineering, Wenzhou Medical University, 270 Xueyuanxi Road, Wenzhou, 325027 China; 4 Key Laboratory of Earth and Planetary Physics, Institute of Geology and Geophysics, Chinese Academy of Sciences, Beijing, 100029 China; 5 Beijing Key Laboratory of Bioelectromagnetism, Institute of Electrical Engineering, Chinese Academy of Sciences, Beijing, 100190 People’s Republic of China; 6 College of Life Science, Huaibei Normal University, Huaibei, 235000 China; 7 Department of Mechanical Engineering, The University of Tokyo, Tokyo, 113-8656 Japan

**Keywords:** Biomaterial, Nanocomposites, Remineralization, Ag-Fe_3_O_4_ hybrid magnetosome, Biosynthesis

## Abstract

**Background:**

Magnetosomes (BMPs) are organelles of magnetotactic bacteria (MTB) that are responsible for mineralizing iron to form magnetite. In addition, BMP is an ideal biomaterial that is widely used in bio- and nano-technological applications, such as drug delivery, tumor detection and therapy, and immunodetection. The use of BMPs to create multifunctional nanocomposites would further expand the range of their applications.

**Results:**

In this study, we firstly demonstrate that the extracted BMP can remineralize in vitro when it is exposed to AgNO_3_ solution, the silver ions (Ag^+^) were transported into the BMP biomembrane (MM) and mineralized into a silver crystal on one crystal plane of Fe_3_O_4_. Resulting in the rapid synthesis of an Ag-Fe_3_O_4_ hybrid BMP (BMP-Ag). The synergy between the biomembrane, Fe_3_O_4_ crystal_,_ and unmineralized iron enabled the remineralization of BMPs at an Ag^+^ concentration ≥ 1.0 mg mL^−1^. The BMP-Ag displayed good biocompatibility and antibacterial activity. At a concentration of 2.0 mg/mL, the BMP-Ag and biomembrane removed Ag-Fe_3_O_4_ NPs inhibited the growth of gram-negative and gram-positive bacteria. Thus using BMP-Ag as a wound dressing can effectively enhance the contraction of infected wounds.

**Conclusions:**

This study represents the first successful attempt to remineralize organelles ex vivo, realizing the biosynthesis of hybrid BMP and providing an important advancement in the synthesis technology of multifunctional biological nanocomposites.

**Graphical abstract:**

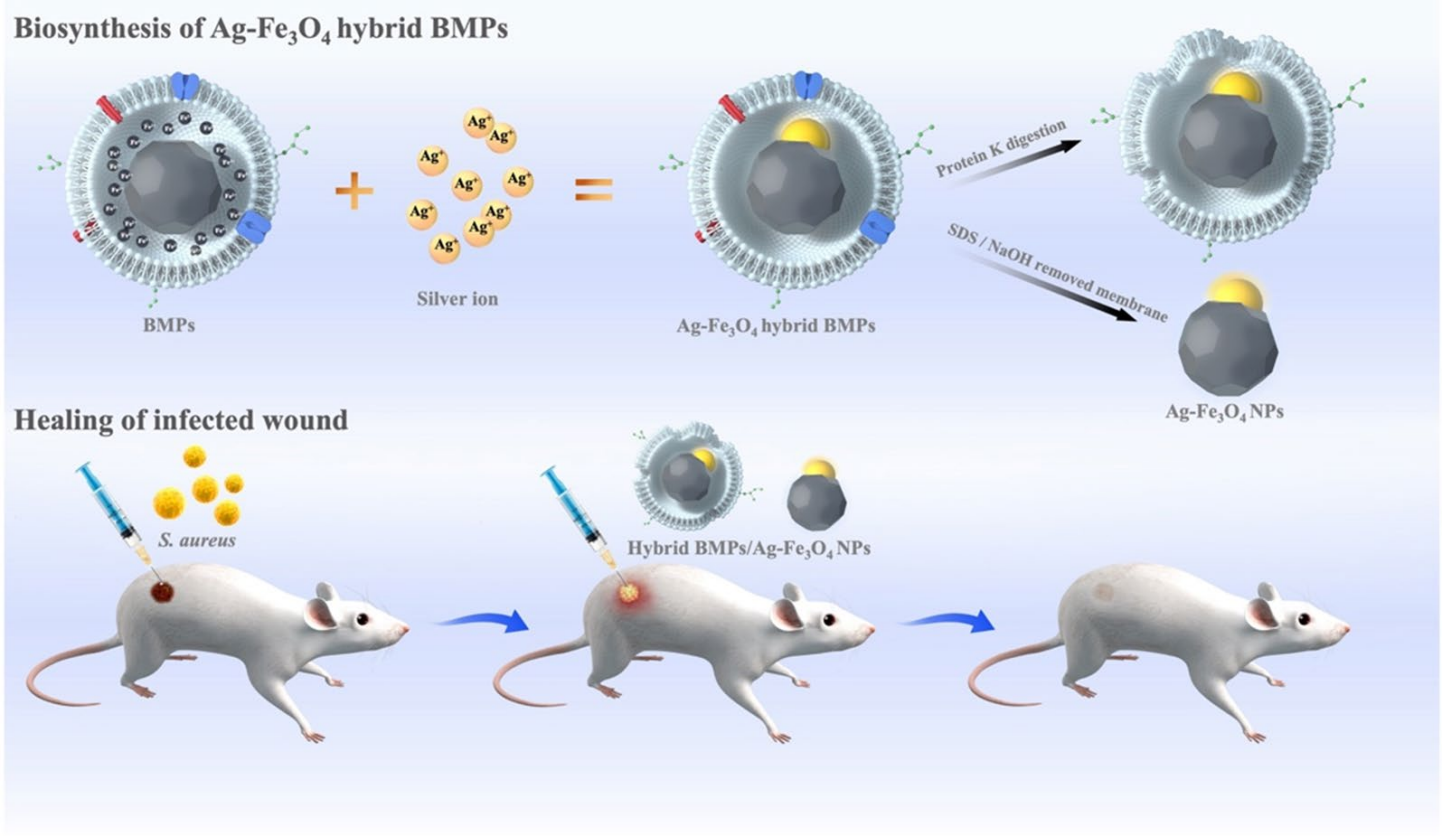

**Supplementary Information:**

The online version contains supplementary material available at 10.1186/s12951-022-01532-4.

## Background

Biomineralization provides specialized biological functions for living organisms, such as mechanical support (e.g., in bones), protection (e.g., in molluscan shells), and mineral storage (e.g., in magnetotactic bacteria magnetosomes). Furthermore, biomineralization plays an important role in biogeochemical element cycles [1]. MTB can absorb iron from the environment and mineralize it to form magnetite nanoparticles (NPs) in its organelle BMPs, contributing significantly to the iron cycle and presenting an elegant example of prokaryote biomineralization [2, 3]. Recently various biological functions of BMPs have been reported, including magnetic navigation [4, 5], iron- and energy-storing abilities [6–8], and the decreased accumulation of free radicals in cells as well as their elimination [9, 10].

In addition, MTB and BMP are ideal candidates for application as microrobots and bionanomaterials, which are widely used in drug delivery, tumor detection and therapy, and immunodetection [11–17], but single-component nanomaterials do not meet the requirements for certain fields. A variety of multifunctional composites have already been synthesized [18–20], primarily based on chemical methods. However, many reagents used during the manufacturing have severely adverse environmental effects and eventually harm human health [21].

Currently, nanoparticles synthesis relates to simple, cost-effective, and eco-friendly methods with multifunctional properties [22, 23]. Green synthesis of nanomaterials includes synthesis at mild pH, pressure, and temperature. It does not entail hazardous substances and avoids addition of external reducing, capping, and stabilizing agents [24, 25]. Green synthesis also facilitate the application of nanomaterials [known as nanobiotechnology) as these nanoproducts are generally biocompatible [25]. A promising method is to biosynthesize nanocomposite via MTB. MTB can synthesize Co, Mn, and Cu-doped BMP in vivo [26–29], which exhibits better magnetic hyperthermia than BMP [30].

Among nanocomposites, two or more functional nanoparticles formed nanohybrids often reveal much better performance in some properties or even generate new features due to the interfacial effect and coupling effect [31–33]. Nevertheless, the biosynthesis of nanohybrids is a challenging. To the best of our knowledge, there have been few reports on nanohybrids biosynthesis [34] and none on asymmetric hybrid metallic nanoparticles by biosynthesis. The situation greatly hinders the application of nanomaterial green synthesis. Therefore, we developed a simple method to biosynthesize asymmetric nanoparticles, via an efficient way of remineralization of ex vivo BMP. We first found that BMP possessing in vitro bioactivity, can transport Ag^+^ into the BMP membrane and mineralized into Ag NPs grown on the magnetite crystal surface, forming an Ag-Fe_3_O_4_ hybrid BMP.

Unsuccessful wound healing caused by bacterial infection is a severe health problem, leading to millions of deaths every year [35]. The bacteria *P. aeruginosa* easily generate biofilm, *E. coli* is representative of gram-negative bacteria, and *S. aureus* is representative of gram-positive bacteria, which can cause several serious infections [36]. Conventional antibiotic has greatly protected the public from bacterial infection, but the large doses of abuse largely increase the drug resistance of bacteria [37]. To solve this problem, engineered materials have been developed to efficiently treat bacterial infections [38–40]. Silver and Fe_3_O_4_ nanoparticles have attracted researchers’ interest in medical devices [41, 42]. Silver nanoparticles (AgNPs] have been widely used in wound healing because of their antimicrobial properties and inhibition of inflammation [43]. Noble-metal-magnetic heterostructures displayed low biotoxicity by preventing the Ag^+^ release rate from the cathodic Ag. Therefore, we further explored the potential of the hybrid BMP to treat bacterial infections efficiently.

## Results and discussion

### BMP production and purification

**Fig. 1:**
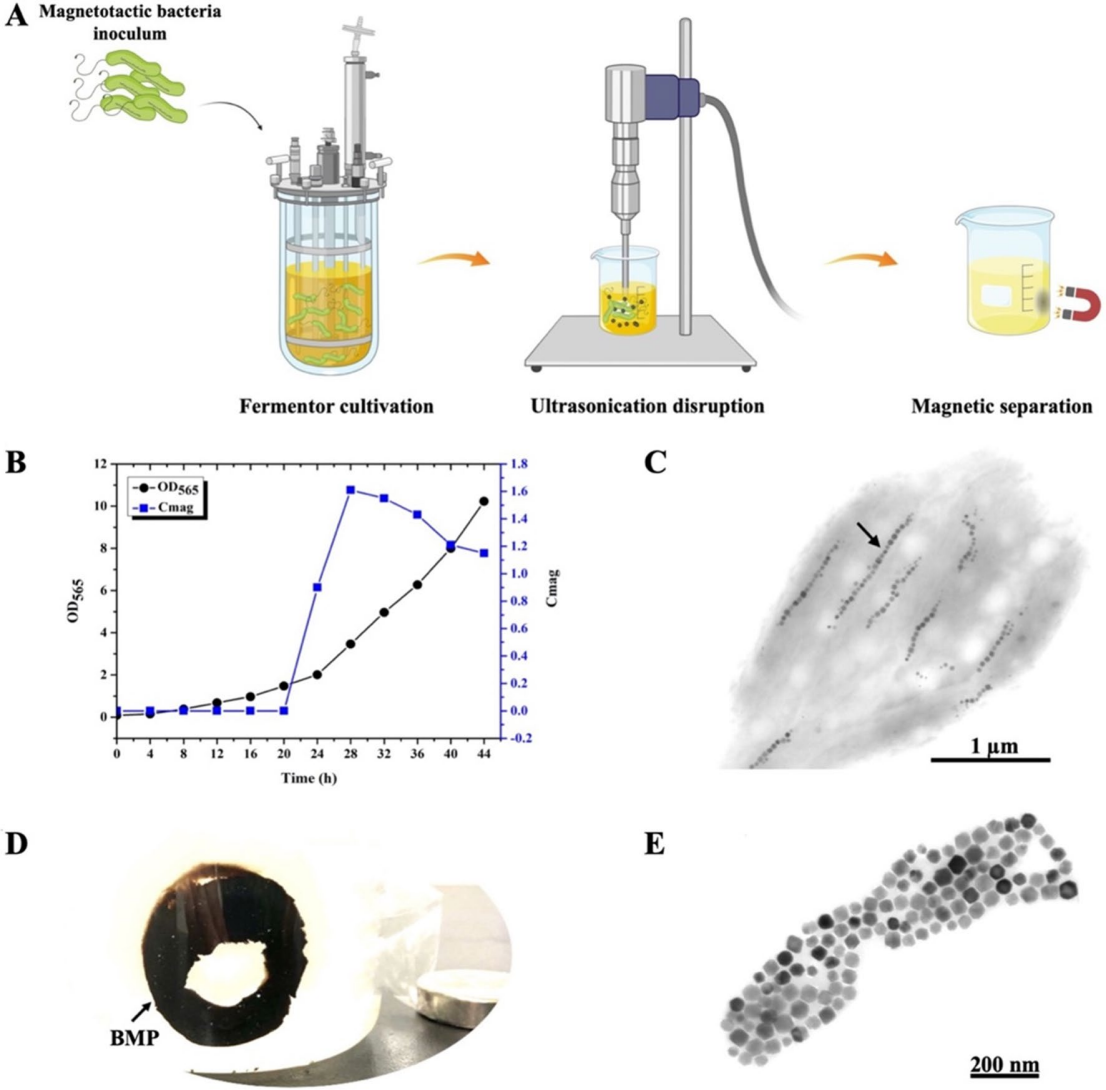
Production and purification of BMPs. **A** Schematics of BMPs production and purification. **B** Cell growth curve and Cmag curve of 42-L fermenter cultured MSR-1. Cell density is estimated by OD_565_. Maximal Cmag value was1.6. Maximal OD_565_ was 10.5. Total dry weight of collected BMP was 6.65 g. **C** Transmission electron microscopy image of the cultured MSR-1. BMPs were arranged in chains in the cell (arrow). **D** Purification of BMPs. Cells were disrupted by ultrasonication, and BMPs (black part) were separated by a magnet. **E** Transmission electron microscopy image of the purified BMPs

BMPs are magnetite nanoparticles synthesized by magnetotactic bacteria that can be achieved through cell disruption and magnetic separation [44, 45]. The preparation process of BMP is shown in (Fig. 1A). Firstly, the magnetotactic bacteria were cultured in a fermenter; then, the cell was collected and disrupted by ultrasonication, and the magnet separated the BMP. Compared with other magnetotactic bacteria, *M. gryphiswaldense* MSR-1 is more suitable for fermenter cultivation. The cell and BMP yield was 2–threefold higher than those of *Magnetospirillum magneticum* AMB-1 and *Magnetospirillum magnetotacticum* MS-1 [32].

A fed-batch culture of MSR-1 was conducted in a 42-L fermenter for production of BMP. Cell density (OD_565_) and magnetic response (Cmag value) were detected in the culture process. After 44 h of culture, OD_565_ and Cmag reached approximately 10.5 and 1.2, respectively (Fig. 1B). Owing to the Cmag value decreasing continuously, MSR-1 fermenter cultivation terminated. The total cell collected was approximately 102.7 g, and the working volume of the fermenter was 30 L; therefore, the cell yield was approximately 3.42 g L^−1^. In fermenter-cultured MSR-1, TEM observation showed that BMPs were arranged in a chain along the cell (Fig. 1C, black arrow).

BMP was purified from the collected cell following several rounds of ultrasonication and magnetic capturing (Fig. 1D, black arrow). The total BMP gained was approximately 6.65 g, and the magnetosome yield was approximately 221.7 mg L^−1^. TEM observation showed that the purified BMP was pure, and there were no protein fragments on BMPs and background (Fig. 1E). BMP was washed 3–5 times in deionized water using an ultrasonic cleaner to remove the remaining PO_4_^3−^ and Cl^−^ from the lysis buffer; then, it was used for Ag^+^ mineralization.

### BMP in vitro remineralization and characterization

**Fig. 2:**
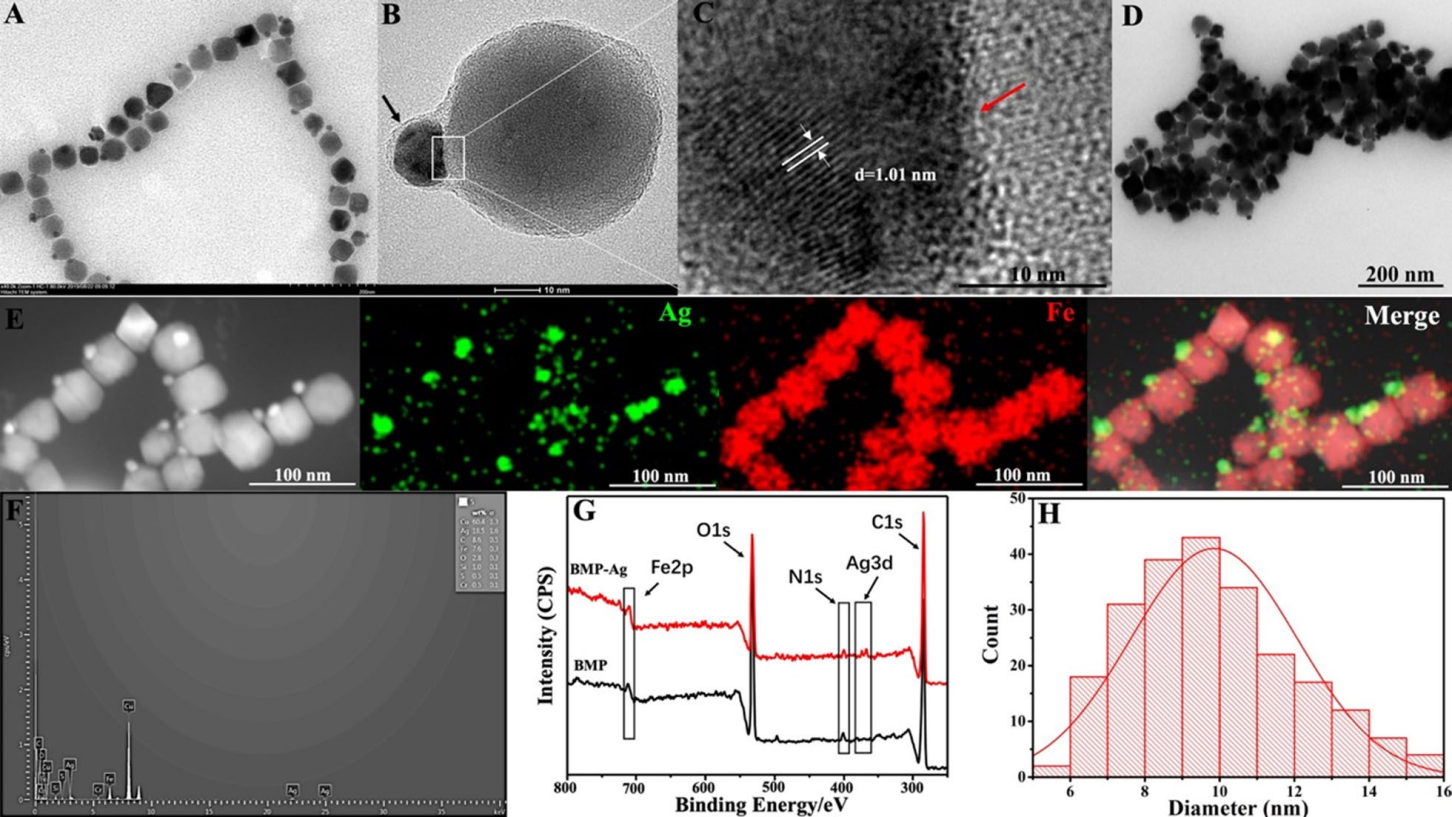
**A** Transmission electron microscopy (TEM) image of BMP after incubation with Ag^+^. BMP was synthesized to form a novel nanocomposite with a Janus-like Ag-Fe_3_O_4_ core. **B**, **C** High-resolution transmission electron microscopy (HTEM) image of hybrid BMP and the interface between Ag and Fe_3_O_4_ crystals. Hybrid BMP was coated by an outer biomembrane (black arrow). Ag and Fe_3_O_4_ lattice were staggered with each other at the interface and formed a whole Ag-Fe_3_O_4_ core. **D** TEM image of the hybrid BMP after removing the biomembrane, Ag NPs did not separate from the Fe_3_O_4_ crystals. **E** HAADF-STEM image and element mapping of Fe and Ag in the hybrid BMP. **F** EDS spectra of Ag NPs of hybrid BMP. **G** XPS patterns of Ag-Fe_3_O_4_ hybrid BMP and BMP. Corresponding particles size distributions of (**H**) Ag NPs of hybrid BMP

The purified BMP was incubated with AgNO_3_ solution. BMP possessed in vitro bioactivity; it transported Ag^+^ into the BMP membrane and was remineralized into a Ag-Fe_3_O_4_ hybrid BMP (Fig. 2A, B). The lattices of Ag and Fe_3_O_4_ were staggered at the interface (Fig. 2C read arrow), which allowed electron transfer in each component and effectively modulated the physical and chemical properties of the BMP. When the outer biomembrane of the hybrid BMP was removed, the Ag NPs did not separate from the Fe_3_O_4_ crystals (Fig. 2D). In Co-, Mn-, and Cu-doped BMP, metal elements are always positioned near the surface of BMP, rather than in the core, and these cannot form crystals [24]. In this study, Ag^+^ was successfully mineralized into Ag crystals on the plane of Fe_3_O_4_ crystals, thereby realizing the in vitro biosynthesis of multifunctional heterogeneous hybrid BMPs.

The elemental composition of the hybrid BMP was further confirmed by high-angle annular dark-field scanning transmission electron microscopy (HAADF-STEM), energy-dispersive spectroscopy (EDS), and X-ray photoelectron spectroscopy (XPS). HAADF-STEM Ag and Fe elemental mapping indicated that the NPs synthesized on the Fe_3_O_4_ crystal plane were Ag NPs (Fig. 2E). EDS analysis of the Ag NPs of the hybrid BMP also demonstrated that excluding most Cu elements from the sample rod, followed by Ag elements, at ~ 18.5% (Fig. 2F). XPS results showed that compared with BMP, Ag-Fe_3_O_4_ hybrid BMP exhibited characteristic Ag diffraction peaks (Fig. 2G). The statistical diameter distributions of the Ag NPs of the hybrid BMP are shown in Fig. 2H. The distribution of Ag NPs was narrow and ranged from 5 to  16 nm and mainly concentrated at 9–10 nm; the average diameter was ~ 10 nm (Fig. 2H). Throughout the remineralization process of BMP, except for the AgNO_3_ solution, no other elements were added. Therefore, the synthesized NPs on the Fe_3_O_4_ crystal were most likely Ag NPs, as confirmed by the HAADF-STEM, XPS, and EDS results.

### BMP remineralization conditions

Biomineralization always occurs under particular physical and chemical conditions. The factors that affect BMP remineralization were evaluated by changing the AgNO_3_ concentration, incubation time, and temperature. Freshly extracted BMP was washed 3–5 times in deionized water using an ultrasonic cleaner to remove the remaining PO_4_^3−^ and Cl^−^ from the lysis buffer and then incubated with AgNO_3_ solution under different conditions.

**Fig. 3:**
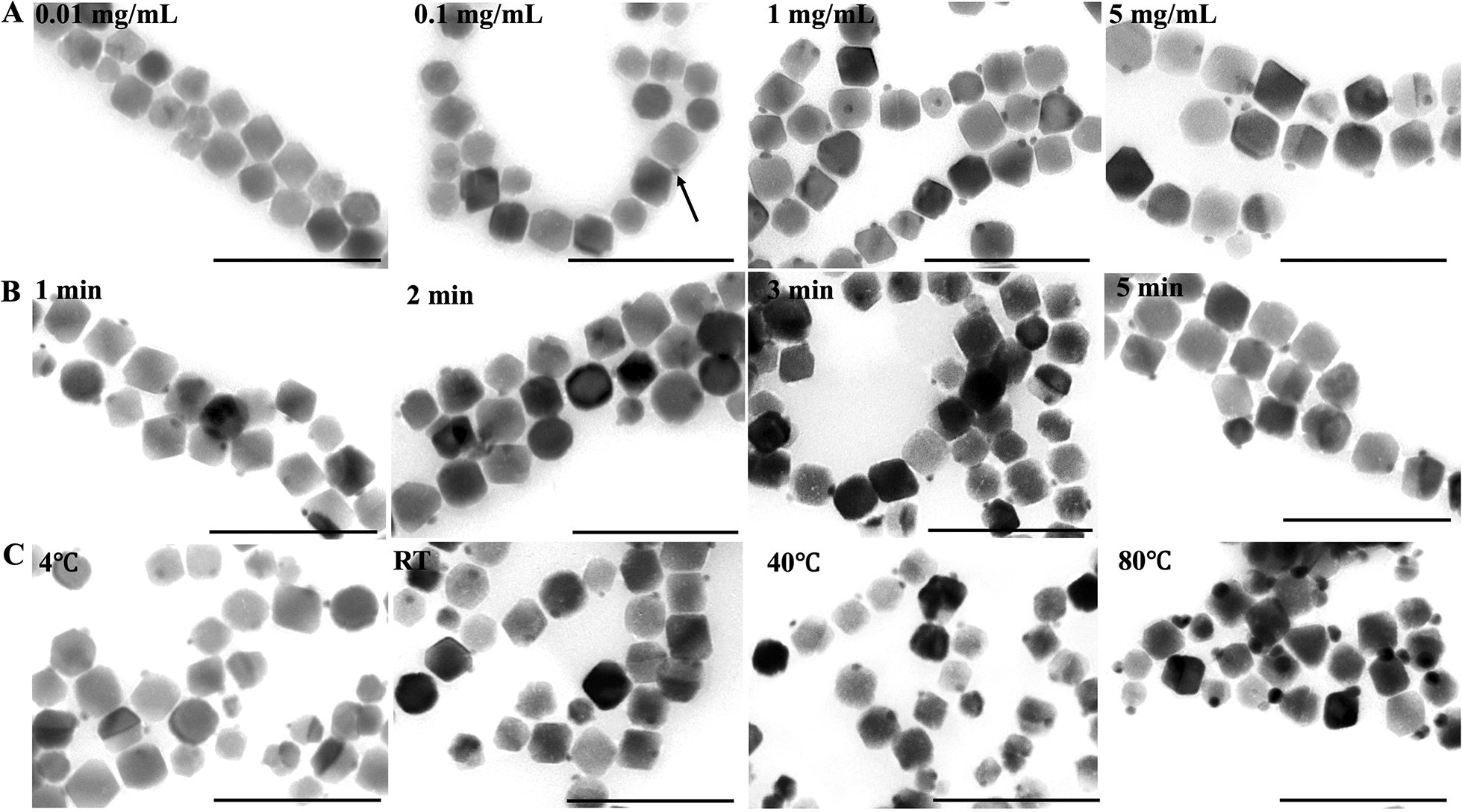
TEM image of BMP after incubation with AgNO_3_ solution at different **A** concentrations, **B** times, and **C** temperatures (bar, 200 nm). Unique conditions necessary for BMP remineralization were an AgNO_3_ concentration ≥ 1.0 mg mL^−1^ and an incubation time ≥ 1 min

A small amount of BMP could mineralize into the Ag-Fe_3_O_4_ hybrid BMP at a AgNO_3_ concentration of 0.1 mg mL^−1^ (Fig. 3A, black arrow). When the concentration increased to 1.0 mg mL^−1^, all BMPs were remineralized ( Fig. 3A). The mineralization process of freshly purified BMP completed rapidly within 1 min, at an AgNO_3_ concentration above 1.0 mg mL^−1^( Fig. 3B). The diameter of the synthesized Ag NPs did not significantly increase with the increasing incubation time. As the BMP storage time increased, the incubation time also increased, and nearly all types of BMP were able to eradicate remineralization within 30 min. BMP was able to remineralize at different temperatures, and the diameter of the synthesized Ag NPs did not significantly increase from 4 to 40 °C ( Fig. 3C). When the temperature was increased to 80 °C, the diameter of the Ag NPs increased by approximately two folds (Additional file 1: Fig. S1). The crystal lattice and functional groups of hybrid BMP synthesize under different parameters were analyzed by X-ray diffraction (XRD) and Fourier transform infrared spectroscopy (FTIR). There are no obvious changes in functional groups, the full width at half maximum decreased as Ag NPs size increased at a reaction temperature of 80 °C (Additional file 1: Fig. S2). These results indicated that the unique conditions necessary for BMP remineralization were an AgNO_3_ concentration ≥ 1.0 mg mL^−1^ and an incubation time ≥ 1 min.

The magnetotactic bacteria MSR-1 cannot synthesize Ag-Fe_3_O_4_ hybrid BMP under these conditions (Additional file 1: Fig. S3) owing to no Ag efflux pump on the MSR-1 cell membrane. Different chemical syntheses Fe_3_O_4_ NPs and commercial magnetic particles cannot synthesize Ag NPs either under these conditions (Additional file 1: Fig. S4). The remineralization just happened on BMPs. Chemical synthesis of Ag-Fe_3_O_4_ hybrids always was a complex process, requiring a catalyst, protective agent, high-temperature, and pressure equipment [46, 47]. The remineralization of BMP at mild pH, pressure, and temperature, did not entail toxic or hazardous substances, and avoided of the addition of external reducing, capping, and stabilizing agents. Thus the remineralization of BMP provided a straightforward and green method to prepare Ag-Fe_3_O_4_ hybrids.

### Antimicrobial activity of hybrid BMPs

**Fig. 4:**
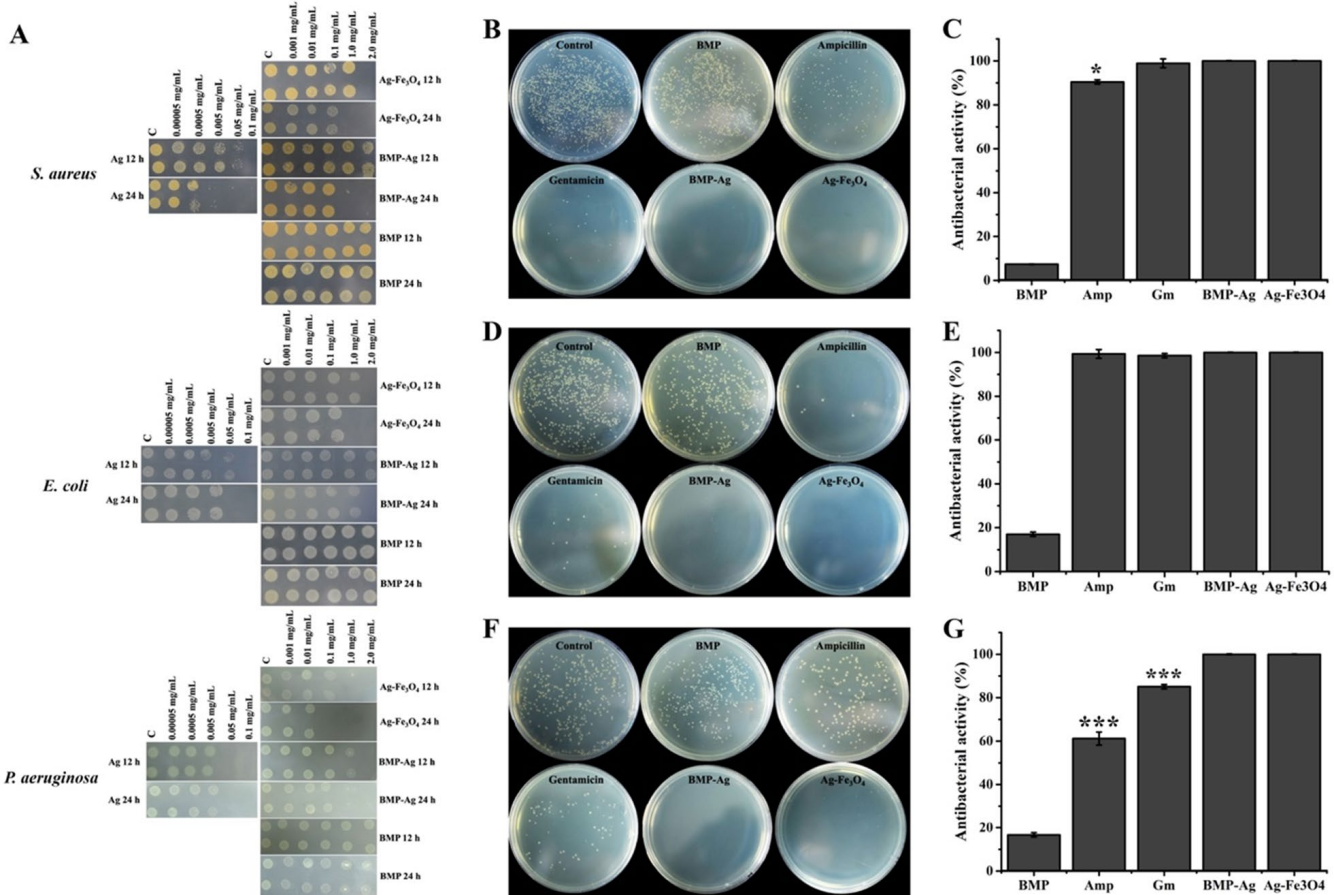
Antibacterial activity of BMP-Ag and Ag-Fe_3_O_4_ NPs. **A** Colony-forming ability of *S. aureus*, *E. coli* and *P. aeruginosa* after exposure to successive dilutions of Ag, Ag-Fe_3_O_4_, BMP-Ag and BMP NPs for 12 h and 24 h at room temperature. At a concentration of 2.0 mg mL^−1^, BMP-Ag and Ag-Fe_3_O_4_ NPs both exhibited antibacterial activity. A low concentration of NPs can inhibit bacterial growth with increased incubated time. Photographs of bacterial colonies formed by *S. aureus *(**B**), *E. coli *(**D**), and *P. aeruginosa *(**F**) after treatment with 2.0 mg mL^−1^ BMP, 100.0 µg mL^−1^ ampicillin, 20.0 µg mL^−1^ gentamicin, 2.0 mg mL.^−1^ BMP-Ag, and Ag-Fe_3_O_4_ NPs. Antimicrobial activity statistics of BMP, ampicillin, gentamicin, BMP-Ag, and Ag-Fe_3_O_4_ NPs to *S. aureus *(**C**), *E. coli *(**E**), and *P. aeruginosa *(**G**). Comparison with antibiotics ampicillin and gentamicin, BMP-Ag and Ag-Fe_3_O_4_ NPs displayed a one hundred percent antibacterial effect. Data are presented as the mean ± s.d. (n = 3 biological replicates per group) and statistically analysed using the two-sided Student’s t-test: *p < 0.05, **p < 0.01, ***p < 0.001, ****p < 0.0001

Wound infections caused by bacteria leading to unsuccessful wound healing is one of serious health problems. Ag NPs display good antibacterial activity [36] and noble-metal-magnetic heterostructures can prevent Ag^+^ release rate from the cathodic Ag, reducing the cytotoxicity of Ag [48]; therefore, Ag-Fe_3_O_4_ hybrid BMP and biofilm removed hybrid BMP (Ag-Fe_3_O_4_ hybrids) can be an ideal wound dressing materials. First, we detected the activities of hybrid BMP and Ag-Fe_3_O_4_ NPs against bacteria by the spot test, with commercial Ag NPs as the positive control and BMP as the negative control. The bacteria *E. coli* (representative of gram-negative bacteria), *S. aureus* (representative of gram-positive bacteria), and *P. aeruginosa*, which can cause several serious infections, were selected to perform the experiments (Fig. 4A). After 12 h of exposure, Ag-Fe_3_O_4_ NPs at a concentration of 2.0 mg mL^−1^ inhibited the growth of *E. coli*, *S. aureus* and *P. aeruginosa*. The antibacterial activity of Ag-Fe_3_O_4_ NPs increased with time, and the antibacterial effect of 1.0 mg mL^−1^ of Ag-Fe_3_O_4_ NPs was observed following 24 h of exposure. Ag-Fe_3_O_4_ NPs presented comparable results with commercial Ag NPs. However, hybrid BMP displayed antibacterial activity at a concentration of 2.0 mg mL^−1^ after 24 h of exposure; the antibacterial activity was lower than those of Ag-Fe_3_O_4_ and Ag NPs. This may be owing to the biomembrane of hybrid BMP prevent the release rate of Ag^+^. BMP NPs had no inhibitory effect on *E. coli*, *S. aureus* and *P. aeruginosa* even up to the highest test concentration of 2.0 mg mL^−1^. Those results indicated that hybrid BMP and Ag-Fe_3_O_4_ NPs all displayed good antibacterial activity.

The antibacterial activities of BMP-Ag and Ag-Fe_3_O_4_ NPs were compared with commercially available antibiotics ampicillin(Amp) and gentamicin(Gm). BMP-Ag and Ag-Fe_3_O_4_ NPs displayed better antibacterial activity than ampicillin and gentamicin. There are no colonies formed of *S. aureus*, *E. coli*, and *P. aeruginosa* after treatment with BMP-Ag and Ag-Fe_3_O_4_ NPs (Fig. 4B, D, F). The antibacterial activity of BMP-Ag and Ag-Fe_3_O_4_ reached 100%, significantly higher than ampicillin 90% for *S. aureus* (Fig. 4C), and than ampicillin 61% and gentamicin 85% for *P. aeruginosa* (Fig. 4G). Antibiotics are selective in their antibacterial properties, e.g. ampicillin has a low antibacterial activity to *P. aeruginosa* and always requires combined use of antibiotics [49–51]. Nanomaterials possess broad-spectrum antibacterial properties and can inhibit all test pathogenic.

### Biosafety evaluation of hybrid BMPs

**Fig. 5:**
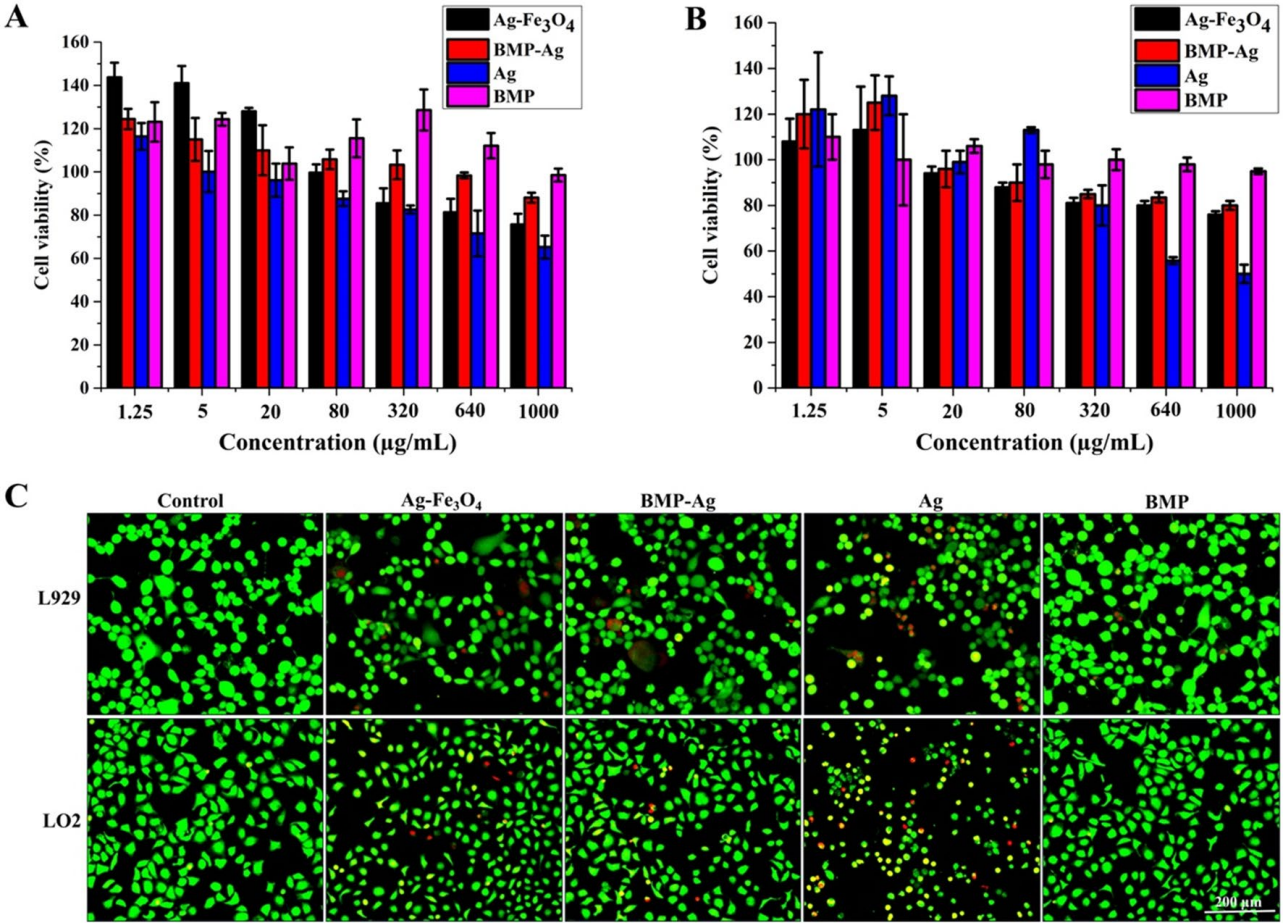
Biosafety evaluation of BMP-Ag and Ag-Fe_3_O_4_ NPs. Cytotoxicity of BMP-Ag and Ag-Fe_3_O_4_ NPs compared with Ag and BMP in different cell lines: **A** L929 and **B** LO2. The cell viability of LO2 and L929 was higher than 75% at the highest concentration of 1000 μg mL^−1^ of BMP-Ag and Ag-Fe_3_O_4_ NPs, which exhibited lower cell cytotoxicity. **C** Fluorescence microscopy images of LO2 and L929 stained with the Calcein-AM/PI kit after treatment with Ag-Fe_3_O_4_, BMP-Ag, Ag, and BMP NPs at the highest concentration 1000 μg mL^−1^

Additionally, the cytotoxicity of BMP-Ag and Ag-Fe_3_O_4_ NPs to mouse fibroblast cell line (L929) and normal human liver cell line (LO2) were detected and compared with those of Ag NPs and BMP using Cell Counting Kit-8 and Calcein-AM/PI kit. As shown in Fig. 5A, B, BMP-Ag and Ag-Fe_3_O_4_ NPs exhibited lower cytotoxicity in the two cell lines than Ag NPs at the same Ag concentration. The cell viability of L929 (Fig. 5A) and LO2 (Fig. 5B) treated with 1000 μg mL^−1^ of BMP-Ag and Ag-Fe_3_O_4_ NPs decreased to approximately 88.1% and 75.7%, and 80.2% and 76%, respectively. The cell viability of L929 and LO2 treated with Ag NPs decreased to approximately 65.2% and 50%, respectively. Even up to the highest test concentration of 1000 μg mL^−1^, BMP was not cytotoxic to the two cell lines. The Calcein-AM/PI cell stain results at the highest test concentration are shown in Fig. 5C; there are fewer red-fluorescent cells (dead cells) in the groups treated with Ag-Fe_3_O_4_, BMP-Ag, and BMP NPs than in those groups treated with Ag NPs (Fig. 5C). These findings indicated that hybrid BMP and Ag-Fe_3_O_4_ NPs have lower cytotoxicity and good biocompatibility to normal mouse and human cell lines and can be used in biomedical applications.

### In vivo wound healing of hybrid BMP

**Fig. 6:**
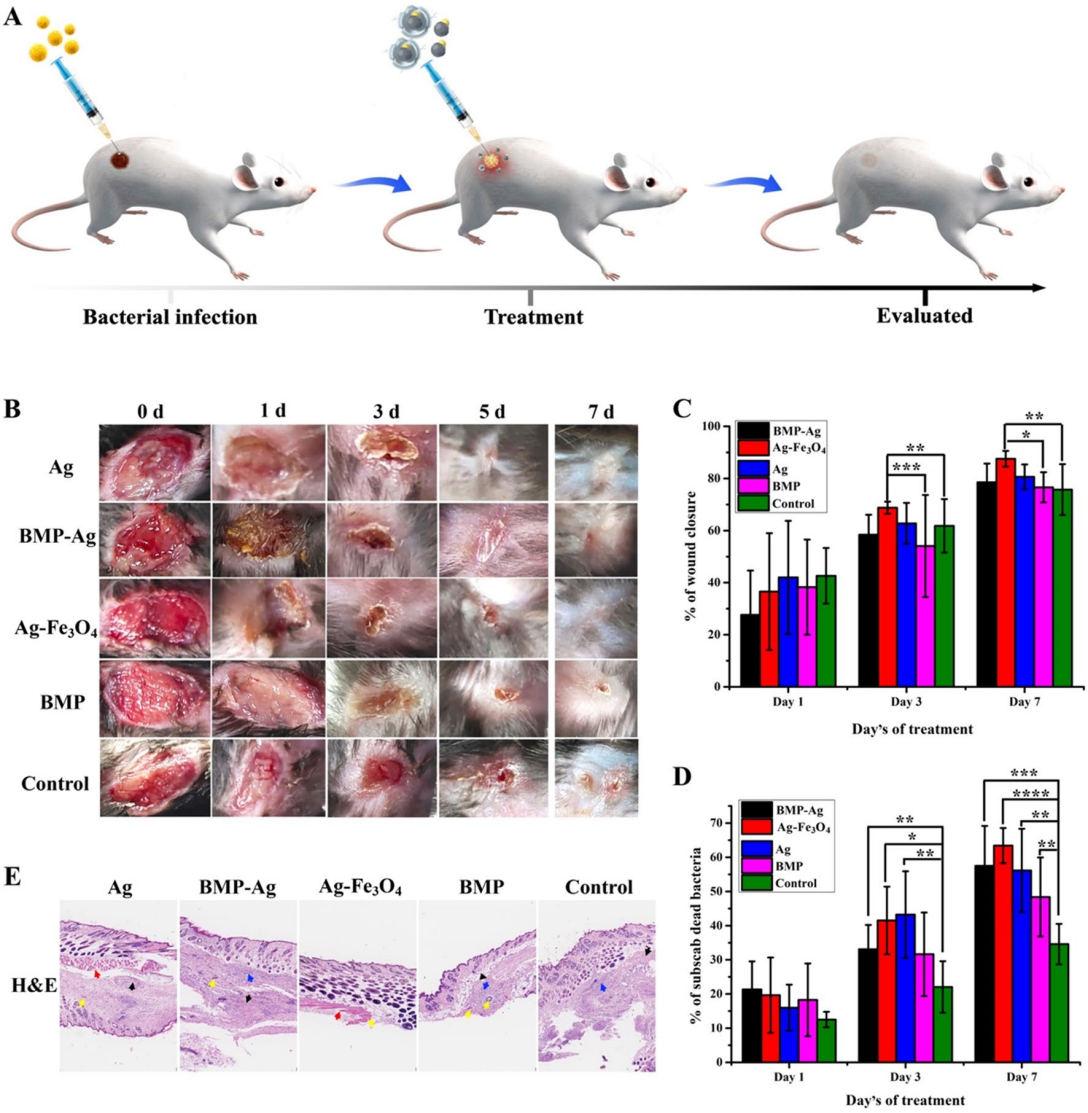
In vivo wound healing of hybrid BMP. **A** Schematic diagram of the experiment against bacterial wound infection in vivo. **B** Wound closure after treatment with BMP-Ag and Ag-Fe_3_O_4_ NPs and comparison with Ag and BMP NPs, with saline solution as the control. On day 7, Ag-Fe_3_O_4_-treated mice wound healed completely and showed enhanced wound contraction compared with other groups. **C**, **D**, different treatment mice wound closure rate (**C**) and bacterial death rate under a scab (**D**) on days 1, 3 and 7. After three days of treatment, the bacteria death rate and the wound closure rate of Ag-Fe_3_O_4_ NPs were significantly higher than those of the control group. **E** Histopathology results show the hematoxylin–eosin (H&E) stained granulation tissues on day 7. Red arrows indicate tissue regeneration; yellow arrows indicate blood vessels; black arrows indicate granulation; blue arrows indicate inflammation. Ag-Fe_3_O_4_ treated infected wound showed obvious tissue regeneration, almost no inflammation compared to control. Data are presented as the mean ± s.d. (n = 3 biological replicates per group) and statistically analysed using the two-sided Student’s t-test: *p < 0.05, **p < 0.01, ***p < 0.001, ****p < 0.0001. *Ag* silver, *BMP* bacterial magnetic nanoparticles, *NP* nanoparticle

Based on the above results, hybrid BMP and relevant Ag-Fe_3_O_4_ NPs showed efficient antibacterial activity and lower cell cytotoxicity; therefore, they are suitable biomaterials against bacterial wound infection. Firstly, a round full-thickness open-excision wound was made on the mice^’^s back and was infected with 500 µL (2 × 10^8^ mL^−1^) *S. aureus*. Then Ag-Fe_3_O_4_ hybrid BMP or Ag-Fe_3_O_4_ NPs were suspended in saline solution, and 80 µL 1.0 mg mL^−1^ Ag-Fe_3_O_4_ /Ag-Fe_3_O_4_ was smeared on the wound to treat the infected wound; the treatment process is shown in schematic diagram Fig. 6A. The therapeutic efficacy was compared with Ag NPs, BMP, and saline (control) treatment groups. The Ag-Fe_3_O_4_ NPs showed enhanced wound contraction compared with Ag, BMP-Ag, BMP, and the control. On day 7, the wounds of Ag-Fe_3_O_4_ NP-treated mice healed almost completely (Fig. 6B). The rate of wound closure was 88%, which was significantly higher than those of the control group with 75%, BMP group with 76%, and Ag NP group with 80% (Fig. 6C). The rates of bacterial death under a scab of the hybrid BMP, Ag-Fe_3_O_4_, Ag, and BMP groups were significantly higher than that of the control group (Fig. 6D). As the treatment time increased, the biomembrane of BMP degraded, showing weak antibacterial activity. Different treated tissue sections were stained with hematoxylin–eosin (H&E) on day 7. The Ag-Fe_3_O_4_ treated infected wound showed obvious tissue regeneration and almost no inflammation compared to control (Fig. 6E). With the antibacterial activity of Ag NPs and Fe_3_O_4_, Ag-Fe_3_O_4_ NPs displayed better therapeutic efficacy.

## Conclusion

To the best of our knowledge, we are the first to report a simple and green method to synthesise BMP-Ag and Fe3O4-Ag NPs through BMP-mediated growth. By mixing BMP with AgNO3 solution of ≥ 1.0 mg/mL, noble metal–magnetic nanohybrids were quickly synthesised within 1 min. Despite the rapid synthesis process, the produced BMP-Ag was stable; therefore, large production of BMP-Ag and Fe3O4-Ag NPs is possible through magnetotactic bacteria fermenter cultivation. BMP-Ag and Fe3O4-Ag NPs displayed good biocompatibility and antibacterial activity and can be used to treat infected wounds. Compared with the chemical synthesis of Ag-Fe3O4 NPs, the synthesis method of BMP-Ag NPs is simple and eco-friendly. BMP-Ag NPs are enveloped by a BMP membrane, which can be easily modified with proteins, nucleic acids, and anticancer drugs and used as a nanocarrier in tumors diagnosis and treatment.

Our findings represent a significant improvement in BMP properties and facilitate the biosynthesis of multifunctional heterogeneous hybrid BMP. BMP acted as an active nano-sized biological reaction kettle in the remineralization process, mineralizing the ions (e.g., Ag^+^) entering the BMP membrane. The proposed approach could lead to the dawn of a new type of biosynthesis technology, potentially mineralizing numerous metal ions or loading drugs into the BMP to synthesize a wider range of multifunctional biomaterials.

## Discussion

Multidrug resistant microorganisms are considered a major health problem worldwide with increasing mortality and morbidity, which raises the need to search for alternative methods of controlling antibiotic-resistant pathogens [52]. Various nanoparticles have made remarkable progress with their promising antibacterial activity in a wide range of bacteria [53]. Among these, silver nanoparticles (Ag-NPs) exert robust, broad-spectrum antimicrobial efficacy through multiple and simultaneous mechanisms and are relatively free of adverse effects. They can increase the permeability of cell membranes, produce reactive oxygen species, interrupt replication of deoxyribonucleic acid, and destroying biofilm by releasing silver ions [54]. Ag-NPs have already been successfully applied in various biomedical and antimicrobial technologies and products used in everyday life [55]. However, the Ag accumulation in the human body and environments showed toxic effects when it deposits to a certain amount [56, 57]. Bacteria can also develop resistance to silver nanoparticles after repeated exposure [58]. Magnetic composites, such as Ag-Fe_3_O_4,_ are a new generation of magnetic antimicrobial NPs, which facilitate local distribution, targeted delivery, and tissue penetration [59, 60]. Targeted delivery reduces bacteria’s exposure time and opportunity to Ag, thereby decreasing the speed by which the pathogen develops resistance and benefiting the removal or retrieval of Ag-NPs [61].

Both Ag-NPs and Fe_3_O_4_ NPs have been intensively studied in wound healing. Besides antibacterial activity, Ag-NPs also possess an anti-inflammatory effect, which made them play a role in the wound healing process by inhibiting the synthesis of tumour necrosis factor [TNF)-α, interferons, and interleukin 1 that involved in inflammatory processes [62]. The functional role of iron in the wound healing process has not been fully understood. Recent interests focused on lactoferrin, an iron-binding glycoprotein secreted from glandular epithelial cells [63]. The protein promotes cutaneous wound healing by enhancing the initial inflammatory phase, and cell proliferation and migration. When treated with lactoferrin, human keratinocytes, fibroblasts and endothelial cells migrate and invade more rapidly in vitro. And the in vivo analysis proved that lactoferrin favourited the closure of skin wounds performed on the mice back [64]. Fe_3_O_4_ NPs were reported to sustained release trace amount of ferric and ferrous ions to their environments [65], thus help to keep the iron homeostasis around wound, and accelerated wound closure, reduced scar width, and enhanced angiogenesis [66]. The composite nanoparticle, Ag-Fe_3_O_4_, simultaneously provided properties of both above NPs in wound healing [65].

MTBs synthesize BMPs and their magnetic crystal cores under mild physiological conditions and strict genetic control. The whole process is a unique and promising platform in the intracellular fabrications of nanomaterials [67]. However, very few materials have been made through this platform. Most studies interests are focused on the direct utilization or surface modification of BMP particles [68]; few have tried to change the composition and shapes of BMP crystals, and only trace amounts of exogenous elements could be incorporate into BMP crystals [29]. Thus, innovative methods are necessary to synthesise engineered BMPs with various compositions and shapes for wider application. Here, we constructed an Ag/Fe_3_O_4_ nanostructure, one of the most important binary systems in recent research hot spots of nanotechnology [69].

**Fig. 7:**
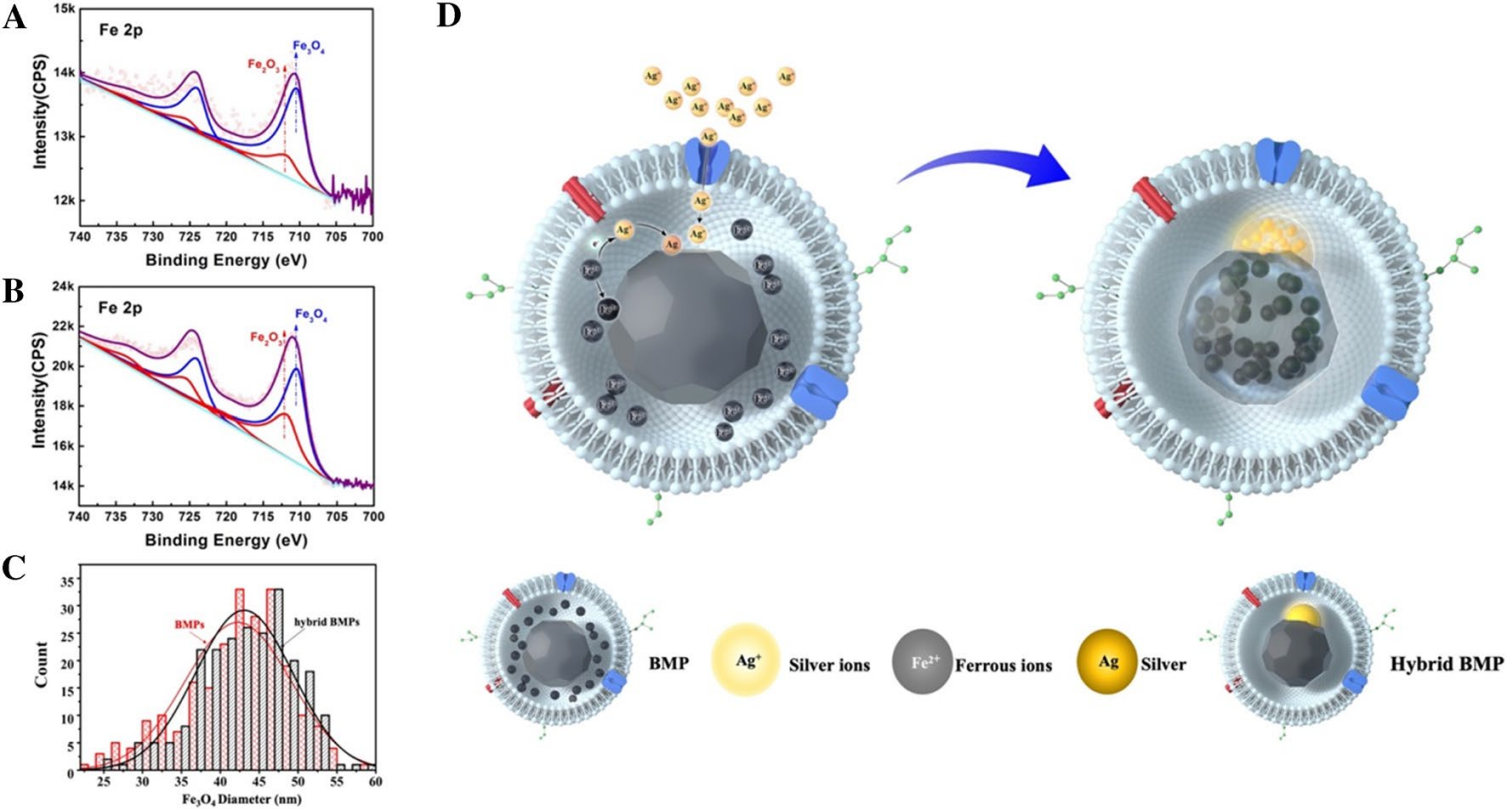
XPS analysis of Fe_3_O_4_ and Fe_2_O_3_ contents of **A** hybrid BMP and **B** BMP. **C** Fe_3_O_4_ diameter statistical distribution of BMP and Ag-Fe_3_O_4_ hybrid BMP. **D** Schematic diagram of BMP-Ag NP synthesis. Ag^+^ is transported into the BMP membrane. The Fe(II) inside the BMP membrane gives an electron to Ag^+^, Ag^+^ is reduced and nucleated, and Ag NPs are formed on one crystal plane of Fe_3_O_4_. Fe(II) that loses an electron was oxidized until biomineralization

Most researchers instinctively thought that purified BMPs were the end products of nanomaterials. However, BMPs contained excess ferrous ions inside MM [23], active proteins and lipids [70]. These components could act as reducing agents and facilitate further synthesis of BMP core crystals even in vitro. For example, ions of noble metals such as silver, gold, and platinum have higher electric potentials than ferrous ions, when transferred into BMP vesicles by diffusion. Thus, they could oxidize ferrous ions and crystalize on the surface of BMP core crystals (Fig. 7D). This process led to the formation of Janus-like BMP-Ag NPs, and the original ferrous ions lost their outer-shell electrons and doped into BMP crystals. Content of Fe_2_O_3_ in the samples increased from 26.2% to 40.9% after remineralization (Fig. 7AB). The average diameter of BMP central crystals was slightly increased from 42.29 ± 6.78 nm to 43.14 ± 6.34 nm (Fig. 7C). However, we found that the Cu/Ag efflux pump *MGMSRv2_1436* protein only exists on BMP membrane (Additional file 1: Table S1) and constructed the *MGMSRv2_1436* mutant strain MSR-Δ*1436* (Additional file 1: Fig. S5). The BMP of MSR-Δ*1436* still mineralized Ag^+^ to form Ag NPs(Additional file 1: Fig. S6), but Ag NPs could not be synthesized by individual Fe_3_O_4_ crystals or BMP membrane(Additional file 1: Fig. S7). The synergy between the biomembrane, Fe_3_O_4_ crystal_,_ and unmineralized iron enabled the remineralization of BMPs. Although BMP displayed bioactivity, it was different from the organism. The mechanisms of BMP remineralization need further investigation.

This work successfully synthesized a new kind of BMPs with heterodimer cores. As the second step of the synthesis was occurred in BMP vesicles, we suggest that BMP was not only just a nanomaterial composite, but also an active biological reaction kettle in nano size.

## Experimental section

*Magnetospirillum gryphiswaldense* MSR-1 fermentation culture: *M. gryphiswaldense* MSR-1 was cultured in a 42-L fermenter (BioFlo110; New Brunswick Scientific, NJ, USA) as described previously [71]. A 5-mL inoculum was activated in 45 mL of fresh sodium lactate medium at 30 °C/100 rpm. Subsequently, MSR-1 was subjected to three sequential transfers, and the third inoculum (3 L) was transferred to a 42-L fermenter. The fermenter had a working volume of 30 L (3 L of inoculum and 27 L of fermentation medium), and the fermentation initial airflow and agitation were set at 0.5 L/min and 100 rpm, respectively. Once the level of dissolved oxygen (dO_2_%) decreased to approximately 15%, airflow was increased to 1.0 L/min. The fermentation dO_2_% level continuously decreased with bacterial growth. Once the dO2% level decreased to approximately 1%, agitation was increased to 10 rpm every 2 h to maintain anaerobic condition. The pH of the fermenter was maintained at 6.8 by automated addition of a lactic acid feeding medium. MSR-1 OD_565_ and magnetic response (Cmag value) were measured during fermentation. Once the Cmag value decreased to approximately 0.8, fermentation was terminated. Cells were collected by centrifugation at 8000 rpm for 10 min and then stored at − 80 °C to purify the BMP.

*Purification of BMPs:* MSR-1 cells were suspended in 50 mmol L^−1^ of phosphate-buffered saline (PBS) with a weight-to-volume ratio of 1:10 and then disrupted by ultrasonication (NingBo Scientz Biotechnology Co., Ltd., China) at 300 W for 30 min. Cellular debris was kept on a neodymium magnet at 4 °C overnight, and the supernatant was emptied. The BMP crude extract was left suspended in 10 mmol L^−1^ of PBS buffer and washed by low-power ultrasonication (power, 200–80 W; operation time, 3 s; inter-arrival, 5 s; total time, 15 min every time) several times. After each wash, the suspended BMP was subjected to permanent magnet adsorption, and the protein concentration in the supernatant was detected on a UV–visible spectrophotometer (UNICO2100; UNICO Instrument Co., Shanghai, China). Pure BMPs were obtained until the protein concentration was < 0.1 mg mL^−1^. BMPs were washed 3–5 times in deionised water using an ultrasonic cleaner to remove the remaining PO_4_^3−^ and Cl^−^ and stored at 4 °C.

*Remineralization of BMPs:* fresh BMPs were mixed with AgNO_3_ solution, incubated at room temperature, and then absorbed by a magnet. After removal of the supernatant, the precipitate was washed 3–5 times in deionized water, obtained Ag-Fe_3_O_4_ hybrid BMP, and stored at 4 °C.

The unique conditions necessary for BMP remineralization were determined by changing AgNO_3_ concentration, incubation time, and temperature.

Janus-like Ag-Fe_3_O_4_ NPs can be made by removing the membrane of Ag-Fe_3_O_4_ hybrid BMP. Hybrid BMP was suspended in 10% sodium dodecyl sulphate and 3-M NaOH mixture, boiled for 10 min and then absorbed by magnet. The precipitate was suspended in deionised water and washed 3–5 times by ultrasonic cleaner; finally, Ag-Fe_3_O_4_ NPs were obtained.

*Physicochemical characterisation of Ag-Fe*_*3*_*O*_*4*_* hybrid BMPs:* hybrid BMPs were characterised by X-ray photoelectron spectroscopy (XPS) (ESCALAB 250Xi, Thermo Fisher Scientific, MA, USA) and dark-field high-angle annular dark-field scanning transmission electron microscopy. The morphology and size of hybrid BMPs were analysed using high-resolution transmission electron microscopy (TEM) (JEOL USA, Inc., MA, USA). BMPs were used as a control.

*Antibacterial activity of hybrid BMPs and Ag-Fe*_*3*_*O*_*4*_* NPs:* the antibacterial activities of hybrid BMPs and Ag-Fe_3_O_4_ NPs against gram-negative (*Escherichia coli*), gram-positive (*Staphylococcus aureus*) and *P. aeruginosa* bacteria were tested by a ‘spot’ assay, as described previously [72]. *E. coli*, *S. aureus*, and *P. aeruginosa* were cultured in 5 mL of a Luria–Bertani (LB) liquid medium at 37 °C 200 rpm^−1^. Cell density OD_600_ was detected in the culture process, and OD_600_ reached approximately 0.5. The cell was collected, washed two times in deionised water, and suspended in 5 mL of ddH_2_O. Subsequently, 100 µL suspensions of *E. coli*, *S. aureus*, and *P. aeruginosa* were incubated with successive dilutions of hybrid BMPs, Ag-Fe_3_O_4_ NPs, and BMP (2.0, 1.0, 0.1, 0.0, and 0.001 mg mL^−1^) and Ag NPs (0.1, 0.05, 0.005, 0.0005, and 0.00005 mg mL^−1^) in a 96-well microtiter plate at room temperature. After 12 and 24 h of incubation, 3 µL of cell suspension was added onto the LB agar medium and incubated at 37 °C in a constant-temperature incubator for 10 h. The number of visible ‘spots’ formed (bacterial colonies) was recorded.

The antibacterial activity compare test of hybrid BMPs and antibiotics were tested by plate count method. The concentrations of antibiotics ampicillin and gentamicin were 100 µg/mL and 20 µg/mL, respectively, for the test, which is the most frequently used concentration in the Luria–Bertani medium. The 100 µL *E. coli*, *S. aureus*, and *P. aeruginosa* were coated one LB solid medium, after treatment with 2.0 mg mL^−1^ hybrid BMPs, Ag-Fe_3_O_4_ NPs and BMP, 0.1 mg mL^−1^ Ag NPs, 100.0 µg mL^−1^ ampicillin, and 20.0 µg mL^−1^ gentamicin, respectively. Then, they were cultured at 37 °C in a constant-temperature incubator for 12 h. The bacterial colonies were photographed and counted. The antibacterial activity (%) was calculated as (Ne-Nc)/Nc × 100, where Ne the number of colonies corresponding to the experiment group, and Nc the number of colonies to the control group.

Hybrid BMPs were pretreated with protein K to digest the protein on the biomembrane. Hybrid BMPs were suspended in protein K solution with a weight ratio of 1:1 and then incubated at 50 °C for 3 h. After digestion, hybrid BMPs were washed 3–5 times in deionized water using an ultrasonic cleaner.

*Wound-healing activity of hybrid BMPs and Ag-Fe*_*3*_*O*_*4*_* NPs:* female C57 mice (20–25 g) aged 6–8 weeks were anaesthetised with 10% chloral hydrate (300 mg/kg) by intraperitoneal injection. Dorsal hair were shaved, and the shaved area was wiped with povidone-iodine. A round full-thickness open-excision wound (8 mm in diameter) was made and then smeared with 500 µL (2 × 10^8^ mL^−1^) of fresh *S. aureus* solution. After 1 day, 80 µL of 0.05 mg mL^−1^ Ag, 1.0 mg mL^−1^ hybrid BMPs, Ag-Fe_3_O_4,_ and BMP were smeared on the wound. The control was smeared with 80 µL of saline solution. Wound healing was recorded by a camera, and the wound area was measured using Photoshop (Adobe Inc., CA, USA). The wound contraction rate was calculated as (original wound area − actual wound area)/original wound area × 100.

The number of bacterial colonies under a scab was counted at days 0, 1, 3 and 7. A 0.01 g of wound edge tissue was cut and homogenised in a micro-homogeniser with 100 mL of saline solution. Subsequently, the homogenate was diluted to 1:10^6^ with saline solution, and 10 µL of the diluent was added onto the LB agar medium and incubated at 37 °C for 12 h. The number of bacterial colonies was counted, and the bacterial death rate under a scab was calculated as (original colonies − actual colonies)/original colonies × 100. All animal protocols were approved by the Biological and Medical Ethics Committee of Beihang University (Approval Number: BM20200087).

Data Analysis and Statistics: The “t-test” was conducted for all statistical analyses. The results are presented as mean ± standard deviation (SD). In all figures, * and ** denote that the p-value is less than 0.05 and 0.01, respectively. All experiments were repeated independently at least three times.

## Supplementary Information

**Additional file 1: Figure S1.** Ag nanoparticle diameter statistical distribution of Ag-Fe_3_O_4_ hybrid BMPs synthesis at room temperature and 80°C. The average diameter of Ag nanoparticles was 9.85±2.22 nm and 17.51±3.14 nm under room temperature and 80℃, which increased almost two fold. **Figure S2.** Functional groups and crystal lattice analysis of BMP-Ag synthesized under AgNO_3_ concentration 1.0 mg/mL, reaction time 1 min, reaction temperature 80°C, and room temperature by FTIR and XRD. The functional groups of BMP-Ag no obvious change under different synthesized parameters. There is a faint diffraction peak at 38.1^o^ of BMP-Ag responding to the (111) crystal plane of face-centered cubic Ag compared with BMP, and the full width at half maximum decreased as the Ag NP size increased at a reaction temperature of 80 °C. **Figure S3.** (A) Magnetotactic bacteria MSR-1 cultured at different concentrations of AgNO_3_ solution. The bacterial growth was inhibited when the AgNO_3_ concentrations >1.0 mg/mL_._ (B) Transmission electron microscopy image of MSR-1 cultured under 1.0 mg/mL AgNO_3_ solution. There is no synthesis of Ag-Fe_3_O_4_ hybrid BMP. **Figure S4.** Transmission electron microscopy image of chemically synthesized Fe_3_O_4_(A), commercial biotinylated magnetic beads(B), and streptavidin magnetic beads(C) and BMPs(D) after incubating with AgNO_3_ solution. Only BMP can mineralize Ag^+^ into Ag nanoparticles. **Figure S5.** Construction of *MGMSRv2_1436* gene deletion mutant. (A) Schematic diagram of the construction of suicide vector pUXcusA. (B) Amplified upstream and downstream fragments of *MGMSRv2_1436* gene and gentamicin resistance cassette gene. (C) Polymerase chain reaction (PCR) amplified the internal gene of *MGMSRv2_1436* (*1436*), gentamicin resistance gene (Gm), upstream and downstream fragments of *MGMSRv2_1436* gene to confirm the screened *MGMSRv2_1436* mutant strain. #: *MGMSRv2_1436* mutant strain, + WT MSR-1 positive control, - ddH2O negative control. There was no *MGMSRv2_1436* gene, but has gentamicin resistance gene, upstream, and downstream fragments in the screened colonies, mean *MGMSRv2_1436* deletion mutant was obtained successfully. (D) PCR amplified the genes (*mamB*, *mamD*, *mamE*, *mamO*, *mamX*, *mamZ*, *mms6*, *feoB*) associated with the magnetosome synthesis. It was confirmed that no genes were lost in the construction process. **Figure S6.** Transmission electron microscopy image of MSR-Δ*1436* BMP after incubating with AgNO_3_ solution. MSR-Δ*1436* BMP still can mineralize Ag^+^ into Ag nanoparticles; it was indicated that Ag^+^ transported into BMP membrane did not base on efflux pump MGMSRv2_1436 protein *in* *vitro* state. **Figure S7.** Transmission electron microscopy image of Fe_3_O_4_ crystal and vesicle(B) of BMP after incubating with AgNO_3_ solution. There was no Ag nanoparticle was synthesis on those two components. It was indicated that remineralization of BMP needs synergy of each element of BMP. **Table S1**. Proteins of only exist on BMP membrane.

## Data Availability

All datasets are available upon reasonable request.
