# Supplementary material for: In vitro magnetosome remineralization for silver-magnetite hybrid magnetosome biosynthesis and used for healing of the infected wound
Source: J Nanobiotechnology. 2022 Aug 6;20:364. doi: 10.1186/s12951-022-01532-4 (PMC9356440; doi:10.1186/s12951-022-01532-4)
Supplement: Supplementary file 1 — Additional file 1: Figure S1. Ag nanoparticle diameter statistical distribution of Ag-Fe3O4 hybrid BMPs synthesis at room temperature and 80°C. The average diameter of Ag nanoparticles was 9.85±2.22 nm and 17.51±3.14 nm under room temperature and 80℃, which increased almost two fold. Figure S2. Functional groups and crystal lattice analysis of BMP-Ag synthesized under AgNO3 concentration 1.0 mg/mL, reaction time 1 min, reaction temperature 80°C, and room temperature by FTIR and XRD. The functional groups of BMP-Ag no obvious change under different synthesized parameters. There is a faint diffraction peak at 38.1o of BMP-Ag responding to the (111) crystal plane of face-centered cubic Ag compared with BMP, and the full width at half maximum decreased as the Ag NP size increased at a reaction temperature of 80 °C. Figure S3. (A) Magnetotactic bacteria MSR-1 cultured at different concentrations of AgNO3 solution. The bacterial growth was inhibited when the AgNO3 concentrations >1.0 mg/mL. (B) Transmission electron microscopy image of MSR-1 cultured under 1.0 mg/mL AgNO3 solution. There is no synthesis of Ag-Fe3O4 hybrid BMP. Figure S4. Transmission electron microscopy image of chemically synthesized Fe3O4(A), commercial biotinylated magnetic beads(B), and streptavidin magnetic beads(C) and BMPs(D) after incubating with AgNO3 solution. Only BMP can mineralize Ag+ into Ag nanoparticles. Figure S5. Construction of MGMSRv2_1436 gene deletion mutant. (A) Schematic diagram of the construction of suicide vector pUXcusA. (B) Amplified upstream and downstream fragments of MGMSRv2_1436 gene and gentamicin resistance cassette gene. (C) Polymerase chain reaction (PCR) amplified the internal gene of MGMSRv2_1436 (1436), gentamicin resistance gene (Gm), upstream and downstream fragments of MGMSRv2_1436 gene to confirm the screened MGMSRv2_1436 mutant strain. #: MGMSRv2_1436 mutant strain, + WT MSR-1 positive control, - ddH2O negative control. There was no MGMSRv2_1436 gene, but has g [file 12951_2022_1532_MOESM1_ESM.docx]

**Supporting Information**

***In Vitro* Magnetosome Remineralization for Silver-Magnetite Hybrid Magnetosome Biosynthesis and used for healing of the infected wound**

Junjie Xu^1,2^†, Shijiao Ma^2^†, Wei Zhang^1^, Lina Jia^1^, Haolan Zheng^2^, Pang Bo^2^, Xue Bai^1^, Hongyan Sun^1^, Lei Qi^3^, Tongwei Zhang^4^, Chuanfang Chen^5^, Feng Li^6^, Fumihito Arai^7^, Jiesheng Tian^2^*, Lin Feng^1^*.


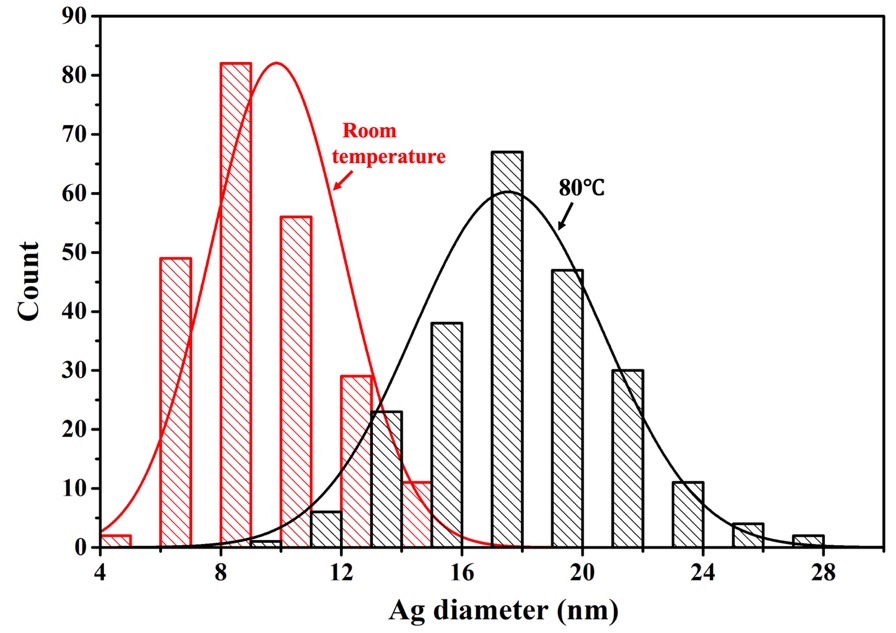


Figure S1. Ag nanoparticle diameter statistical distribution of Ag-Fe_3_O_4_ hybrid BMPs synthesis at room temperature and 80°C. The average diameter of Ag nanoparticles was 9.85±2.22 nm and 17.51±3.14 nm under room temperature and 80℃, which increased almost two fold.

Figure S2. Functional groups and crystal lattice analysis of BMP-Ag synthesized under AgNO_3_ concentration 1.0 mg/mL, reaction time 1 min, reaction temperature 80°C, and room temperature by FTIR and XRD. The functional groups of BMP-Ag no obvious change under different synthesized parameters. There is a faint diffraction peak at 38.1^o^ of BMP-Ag responding to the (111) crystal plane of face-centered cubic Ag compared with BMP, and the full width at half maximum decreased as the Ag NP size increased at a reaction temperature of 80 °C.


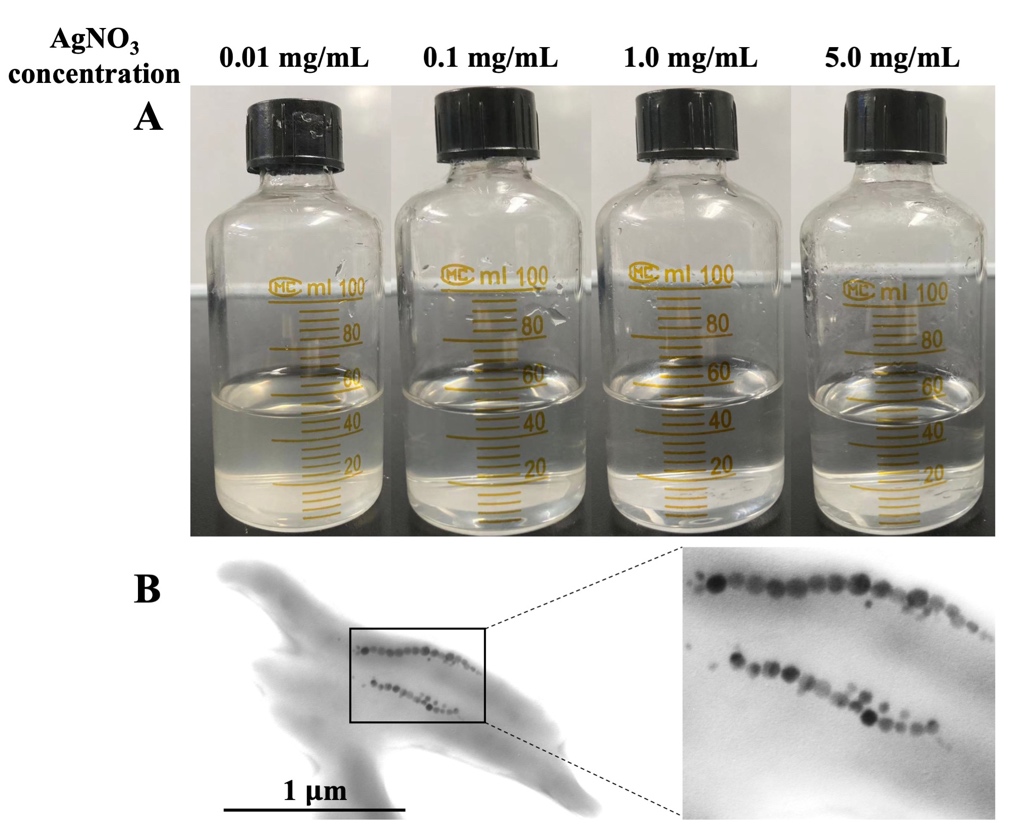


Figure S2. (A) Magnetotactic bacteriaMSR-1 cultured at different concentrations of AgNO_3_ solution. The bacterial growth was inhibited when the AgNO_3_ concentrations >1.0 mg/mL_._ (B) Transmission electron microscopy image of MSR-1 cultured under 1.0 mg/mL AgNO_3_ solution. There is no synthesis of Ag-Fe_3_O_4_ hybrid BMP.


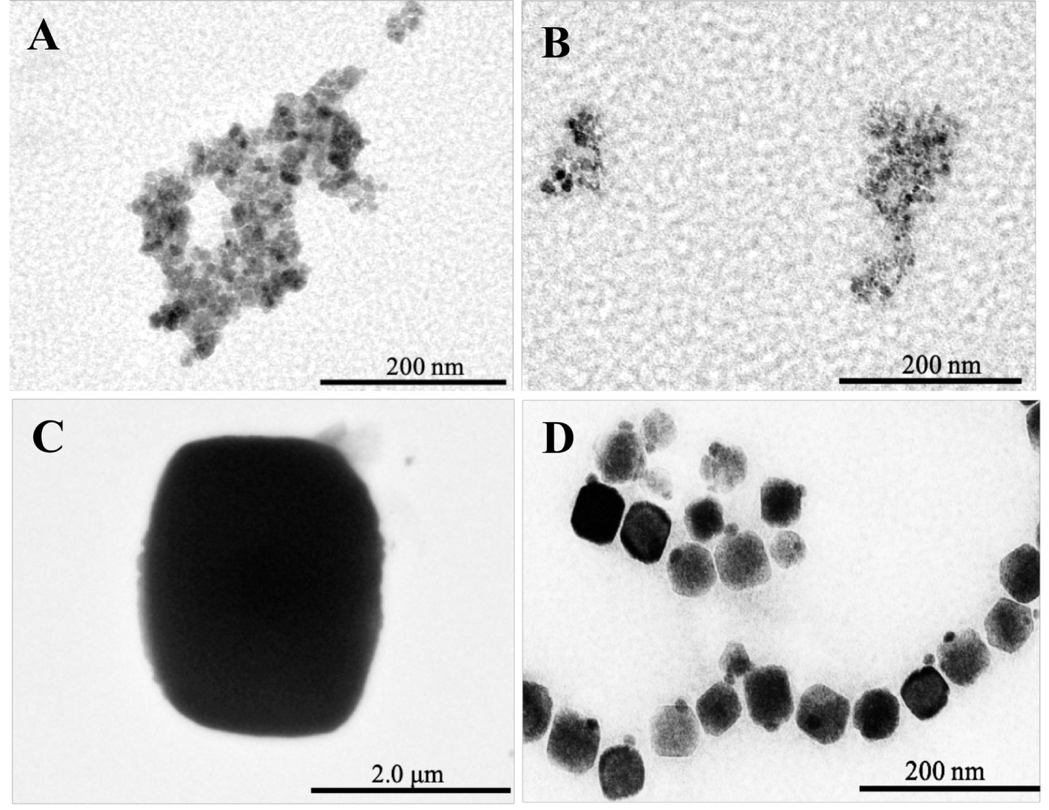


Figure S4. Transmission electron microscopy image of chemically synthesized Fe_3_O_4_(A), commercial biotinylated magnetic beads(B), and streptavidin magnetic beads(C) and BMPs(D) after incubating with AgNO_3_ solution. Only BMP can mineralize Ag^+^ into Ag nanoparticles.


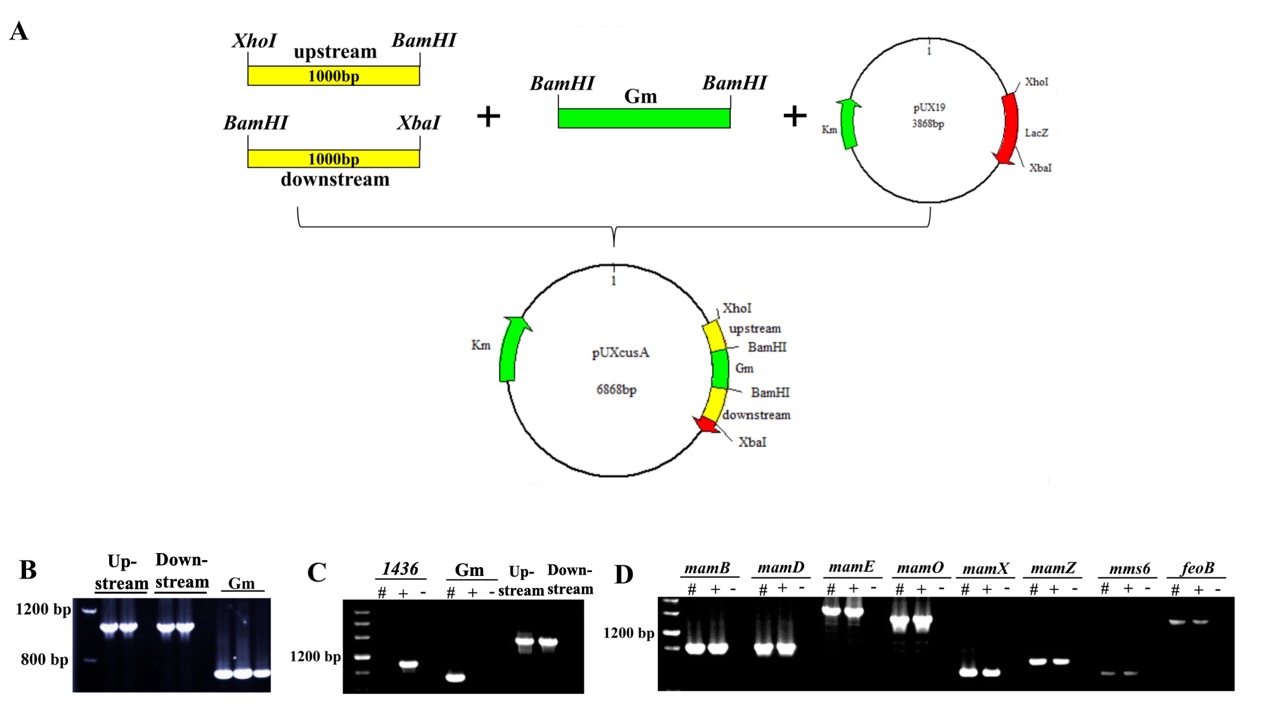


Figure S5. Construction of *MGMSRv2_1436* gene deletion mutant. (A) Schematic diagram of the construction of suicide vector pUXcusA. (B) Amplified upstream and downstream fragments of *MGMSRv2_1436* gene and gentamicin resistance cassette gene. (C) Polymerase chain reaction (PCR) amplified the internal gene of *MGMSRv2_1436* (*1436*), gentamicin resistance gene (Gm), upstream and downstream fragments of *MGMSRv2_1436* gene to confirm the screened *MGMSRv2_1436* mutant strain.#: *MGMSRv2_1436* mutant strain, + WT MSR-1 positive control, - ddH2O negative control. There was no *MGMSRv2_1436* gene, but has gentamicin resistance gene, upstream, and downstream fragments in the screened colonies, mean *MGMSRv2_1436* deletion mutant was obtained successfully. (D) PCR amplified the genes (*mamB*, *mamD*, *mamE*, *mamO*, *mamX*, *mamZ*, *mms6*, *feoB*) associated with the magnetosome synthesis. It was confirmed that no genes were lost in the construction process.


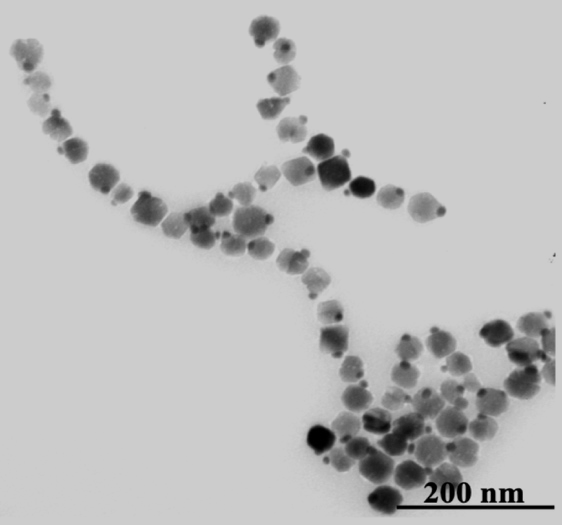


Figure S6. Transmission electron microscopy image of MSR-Δ*1436* BMP after incubating with AgNO_3_ solution. MSR-Δ*1436* BMP still can mineralize Ag^+^ into Ag nanoparticles; it was indicated that Ag^+^ transported into BMP membrane did not base on efflux pump MGMSRv2_1436 protein *in vitro* state.


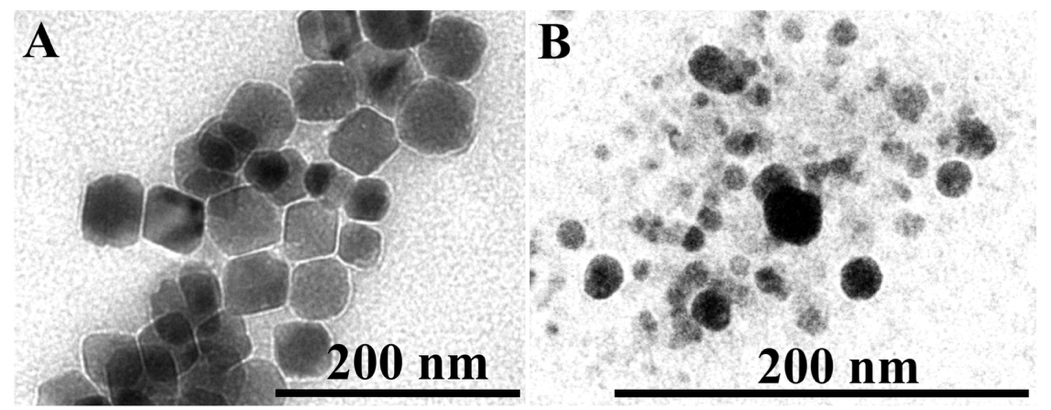


Figure S7. Transmission electron microscopy image of Fe_3_O_4_ crystal and vesicle(B) of BMP after incubating with AgNO_3_ solution. There was no Ag nanoparticle was synthesis on those two components. It was indicated that remineralization of BMP needs synergy of each element of BMP.

Table S1 Proteins of only exist on BMP membrane

**Supplementary experimental section**

**Construction of *MGMSRv2_1436* deletion mutant** : *MGMSRv2_1436* deletion mutant (MSR-*Δ1436*) was constructed by biparental conjugation. The fragments 1000 bp upstream and downstream of *MGMSRv2_1436* were amplified from the MSR-1 genome using primers sets. Gentamicin resistance cassette was amplified from the pUCGm vector. Then the amplified upstream fragment was digested by restriction endonucleases *XhoI* and *BamHI*. The downstream fragment was digested by *XbaI* and *BamHI,* and the Gentamicin resistance cassette was digested by *BamHI*. Upstream, downstream and gentamicin resistance cassettes were ligated into suicide vector pUX19 to form pUXcusA (Figure S3AB). pUXcusA was transformed into *E. coli* S17-1 to construct donor strain. Then pUXcusA was introduced into WT MSR-1 by biparental conjugation. MSR-*Δ1436* was screened and selected by Gm^r^ and Nx^r^ and confirmed by Polymerase chain reaction (PCR). There was no *MGMSRv2_1436* gene, but has gentamicin resistance gene, upstream and downstream fragments in the screened colonies, mean achieved *MGMSRv2_1436* deletion mutant successfully(Figure S3C). The genes associated with magnetosomes synthesis were detected in MSR-*Δ1436* and confirmed that no genes were lost in the construction process(Figure S3D).

**Extraction vesicle and Fe_3_O_4_ crystal of magnetosome:** To detect a different component of BMP effect on Ag^+^ mineralization, vesicle and Fe_3_O_4_ crustal of BMP were extracted, respectively. Magnetotactic bacteria could survive empty synthesis magnetosomes when cultured in iron-poor condition(1). We constructed *in vivo* biotinylated magnetosome in MSR-1 as described previously(2). The constructed recombinant strain (MSR-∆F-BF) that synthesized biotinylated magnetosome were cultured without iron medium for 24 h—then collected by centrifugation at 8000 rpm for 10 min. The cell was suspended in 10 mmol/L of PBS buffer and lysed by ultrasonication (NingBo Scientz Biotechnology Co., Ltd., China) at 100 W for 30 min. We extracted biotinylated vesicle from cell lysis solution based on particular biotin bind with streptavidin. Streptavidin magnetic beads were purchased from Thermo Fisher and incubated with a cell lysis solution. Then biotinylated BMP vesicle was extracted according to the instructions.

Fe_3_O_4_ crustal was prepared by suspended 1 mg BMP in 10 mL 10% sodium dodecyl sulfate (SDS) and 3 mol/L NaOH mixture, then boiled for 15 min, magnetic separation, and 10 mL ddH_2_O washed 3-5 times.

**References**

1. Komeili A, Vali H, Beveridge TJ, Newman DK. Magnetosome vesicles are present before magnetite formation, and MamA is required for their activation. Proc Natl Acad Sci U S A. 2004;101(11):3839-44.

2. Maeda Y, Yoshino T, Takahashi M, Ginya H, Asahina J, Tajima H, et al. Noncovalent immobilization of streptavidin on in vitro- and in vivo-biotinylated bacterial magnetic particles. Appl Environ Microbiol. 2008;74(16):5139-45.
